# Supplementary material for: Content-rich biological network constructed by mining PubMed abstracts
Source: BMC Bioinformatics. 2004 Oct 8;5:147. doi: 10.1186/1471-2105-5-147 (PMC528731; doi:10.1186/1471-2105-5-147)
Supplement: Additional File 2 — The original results of the above study (non-essential files are deleted to keep the file size under the limit set by BMC bioinformatics). [file 1471-2105-5-147-S2.bz2 › chilibotAdditionalFile2/dip10/385ID9252374E9850/html/THPO_MPL.html]

 


 **THPO** and **MPL** 
  
Found 539 abstracts in PubMed, retrieved 10.  
 

 What does Google say? 
 PDF only 
| .edu only 

---

**Interactive relationship** (e.g. stimulation, inhibition, etc)

**Inhibitory relationship**- Our case report suggests that AMT in patients with SSc could be mediated by the anti c  **Mpl**   [ **MPL** ]  antibody, which functionally blocks an interaction between thrombopoietin  [ **THPO** ]  and c  **Mpl**   [ **MPL** ] .  Ref: 12794833 Arthritis Rheum, 2003
**Stimulatory relationship**- Interestingly, decreased c  **MPL**   [ **MPL** ]  and elevated PRV 1 were also observed in patients with hereditary thrombocythemia HT who carry a mutation in the thrombopoietin  [ **THPO** ]  TPO  [ **THPO** ]  gene.  Ref: 12730106 Blood< MedlineTA>Blood, 2003
- Although it is clear that thrombopoietin  [ **THPO** ]  is the primary regulator of thrombopoiesis, several lines of evidence indicate that the hormone affects multiple aspects of hematopoiesis the in vivo administration of TPO  [ **THPO** ]  increases marrow levels of erythroid, myeloid, and megakaryocytic progenitor cells and its genetic elimination or that of its receptor c  **mpl**   [ **MPL** ]  reduces the numbers of these cells.  Ref: 12799280 Ann N Y Acad Sci< MedlineTA>Ann N Y Acad Sci, 2003
**Neutral relationship**- Thrombopoietin  [ **THPO** ]  TPO  [ **THPO** ]  is a hematopoietic cytokine that regulates megakaryocytosis and thrombocytosis by binding to its receptor c  **Mpl**   [ **MPL** ] .  Ref: 12841634 Mol Cell Biochem, 2003

**Non-interactive relationship** (e.g. studied together, co-existance, homology, etc.)

- A corollary of this finding is that TPO  [ **THPO** ]  or the c  **Mpl**   [ **MPL** ]  receptor is a candidate for involvement in the genesis of chronic myeloproliferative diseases MPD, disorders of hematopoietic stem cells.  Ref: 12682875 Semin Hematol, 2003
- METHODS Megakaryocyte progenitor cell subpopulations from cord blood CB and mobilized peripheral blood PBSC were expanded in vitro in the presence of  **mpl**  ligand  [ **THPO** ] .  Ref: 12691920 Exp Hematol, 2003
- Her serum was strongly positive for anti c  **Mpl**   [ **MPL** ]  antibody, and IgG fraction purified from her serum inhibited thrombopoietin  [ **THPO** ]  dependent cell proliferation in vitro.  Ref: 12794833 Arthritis Rheum, 2003
- Like CAMT patients, mice lacking the TPO  [ **THPO** ]  receptor c  **Mpl**   [ **MPL** ]  demonstrate a major reduction of early hematopoietic progenitor cells of all lineages.  Ref: 12799278 Ann N Y Acad Sci, 2003
- Recognition of defective platelet thrombopoietin  [ **THPO** ]  receptor  **Mpl**  expression and overexpression of the PRV 1 gene in PV neutrophils has led to studies demonstrating the potential use of these molecular abnormalities as diagnostic markers for PV and for risk stratifying patients with ET for disease conversion to PV or IMF.  Ref: 12682874 Semin Hematol, 2003
- Thus, TPO  [ **THPO** ]  and  **Mpl**  may prove to be effective targets for both diagnosis and therapeutic management of MPD.  Ref: 12682875 Semin Hematol, 2003
- we show a similar modulation by TGF beta1 of a fourth receptor the TPO  [ **THPO** ]  receptor  **MPL** .  Ref: 12725536 Oncol Res, 2003
- Anti c  **Mpl**   [ **MPL** ]  thrombopoietin  [ **THPO** ]  receptor autoantibody induced amegakaryocytic thrombocytopenia in a patient with systemic sclerosis.  Ref: 12794833 Arthritis Rheum, 2003
- Here we describe a rare case of SSc accompanied by thrombocytopenia and megakaryocytic hypoplasia, in which autoantibody against thrombopoietin  [ **THPO** ]  receptor c  **Mpl**   [ **MPL** ]  was detected.  Ref: 12794833 Arthritis Rheum, 2003
- Because thrombopoietin  [ **THPO** ]  TPO  [ **THPO** ]  and its receptor, c  **Mpl**   [ **MPL** ] , regulate primitive hematopoietic populations, including bone marrow hematopoietic stem cells, we investigated whether TPO  [ **THPO** ]  acts on the hemangioblasts that derive from differentiation of embryonic stem cells in vitro.  Ref: 12743322 Stem Cells, 2003
